# Effect of epidermal growth factor receptor status on the outcomes of patients with metastatic gastric cancer: A pilot study

**DOI:** 10.3892/ol.2013.1662

**Published:** 2013-11-06

**Authors:** K. AYDIN, S.K. OKUTUR, M. BOZKURT, I. TURKMEN, E. NAMAL, K. PILANCI, A. OZTURK, Z. AKCALI, G. DOGUSOY, O.G. DEMIR

**Affiliations:** 1 Department of Medical Oncology, Istanbul Bilim University, Istanbul, Turkey; 2 Department of Pathology, Istanbul Bilim University, Istanbul, Turkey; 3 Department of Pathology, Gayrettepe Florence Nightingale Hospital, Istanbul, Turkey

**Keywords:** epidermal growth factor receptor, gastric cancer, prognosis

## Abstract

The expression of epidermal growth factor receptor (EGFR) has been linked to clinical outcome in several solid tumors. However, the clinical significance of EGFR (c-erbB1) in gastric cancer remains unclear. The present study was designed to detect the clinical implications of EGFR in the Turkish population. Paraffin-embedded tissue microarrays containing gastric cancer tissue were obtained from 30 patients. EGFR expression was detected using immunohistochemistry. The correlation of this biomarker to the clinicopathological features and survival of patients with gastric cancer was studied. The overall positivity rate of EGFR was 63.3%. EGFR expression was significantly correlated with an improved progression-free survival (PFS) and overall survival (OS) rate (P=0.039 and 0.01, respectively). EGFR expression is a good prognostic marker for patients with gastric cancer.

## Introduction

Gastric carcinoma is the fourth most common cancer, with 1 million new cases per year, and the second leading cause of cancer-related mortality worldwide (1). A diagnosis often occurs in the advanced stages and thus the prognosis is usually poor. Although new cytotoxic therapies are being tested (2–4), the median overall survival (OS) is ~10 months. Encouraging results from targeted therapies in other cancers have led to an interest in such therapies and the identification of biomarkers in gastric cancer. To date, it has been shown that targeted therapies are useful in gastric cancer in the form of treatment using the anti-HER2 antibody, trastuzumab, which is used to treat patients with HER2 overexpression. HER2 is a member of the epidermal growth factor receptor (EGFR) family (5). EGFR is a member of the tyrosine kinase receptor family, which consists of four structurally similar, but functionally varied receptors, including erbB1 (HER1/EGFR), erbB2 (HER2/neu), erbB3 (HER3) and erbB4 (HER4). All these transmembrane receptors contain intrinsic kinase activities and are activated by modified tyrosine residues. It is believed that the aberrant activation of the signaling pathway contributes to tumorigenic events, including increased cellular proliferation, prevention of apoptosis, tumor cell invasion and metastasis (6). In numerous studies, it has been shown that the EGFR status is an independent prognostic factor in various tumor types (7). The present study aimed to evaluate the prognostic significance of EGFR expression in patients with advanced gastric cancer.

## Patients and methods

### Patients

A retrospective cohort study was conducted in Istanbul Bilim University, (Istanbul, Turkey) involving 40 patients with gastric adenocarcinoma who were diagnosed using a histological stomach tissue sample examination by the Department of Pathology. The patients were followed by the Medical Oncology Clinic of Istanbul Bilim University between 2008 and 2011. A total of eight patients were excluded from the study, one due to a second primary tumor, four due to the presence of stage II disease and three due to their pathology blocks being unavailable. All patients that were included in the study already had metastatic disease or developed it during the follow-up period. At the time of the present analysis, 18 of the patients (60%) had succumbed to their diseases. This study was approved by the ethics committee of Istanbul Bilim University. Informed consent was obtained from the patients.

### Pathology and immunohistochemistry (IHC)

The formalin-fixed paraffin-embedded tumor samples were evaluated for EGFR protein expression using IHC. Sections (2-μm thick) were cut from the paraffin embedded tissue blocks, fixed with 10% formaldehyde and fixed on marked slides. The slides were maintained in an incubator at 56°C overnight and deparaffinized by being soaked twice in Xylene for 15 min and in 96% alcohol three times for 5 min. The slides were finally rehydrated with distilled water. Distilled water was added to Decloaking Chamber Plus (Biocare Medical, Concord, CA, USA); a system with a cooling fan and high pressure for a more rapid and homogenous antigen retrieval. The slides were placed in a 10% citrate buffer (pH, 6.0) solution. The slides were then placed in the chamber by washing in an antigen recovery solution for 30 sec at 125°C and 10 sec at 90°C and were allowed to cool to room temperature for 10–20 min. The edges of the slides were dried and the tissue boundaries were drawn using a Pap pen (Abcam, Cambridge, MA, USA) subsequent to being washed in Tris-buffered saline plus Tween 20 (TBST). Blockage of the endogenous peroxidase activity was performed by dripping 3% hydrogen peroxide (H_2_O_2_) on each tissue sample and then placing them in a moist environment for 10 min. The samples were then soaked in phosphate-buffered saline (PBS) for 5 min, subsequent to being washed in distilled water. EGFR antibodies (1:60, Clone: EGFR.25, Produnt code: NCL-EGFR-384; Leica-Novocastra, Solms, Germany) were added to the slides, which were shaken in order to remove excess liquid from the slides, placed into an incubation vessel and stored for 2 h. The slides were then placed in staining jars that contained PBS. The slides were incubated with the secondary antibody (multispecies ultra streptavidine detection system, HRP, Zymed, MA, USA) and streptavidine-biotin complex (Zymed) was administered on the slides, which were incubated again for 20 min. Streptavidin was dripped onto the slides following PBS4, used to maintain a constant pH, and allowed to dry for 20 min. A solution was prepared using 1 ml distilled water, one drop AEC buffer, two drops AEC chromogen and one drop concentrated H_2_O_2_, which was then added to the slides. The opposite coloring was performed using Mayer hematoxylin in a 2-min application. The slides were kept in a water-based closing medium (aquesmount, Scytec, Logan, UT, USA) following the completion of the empurpling process in tap water.

All the slides that were stained using hematoxylin and eosin (HE) and IHC were examined by two pathologists who were experienced in gastrointestinal cancers. In all the cases, the antibodies were evaluated separately in the invasive tumor and surrounding gastric tissue slides. Cytoplasmic and membranous staining was obtained for EGFR. Placental tissue was used as a control for EGFR. A scoring system was used, as the staining intensities were variable. The result that was obtained semi-quantitatively by multiplying the percentage of the positively-stained cells and the staining intensity was recorded as the immunoreactive score (IRS). The staining intensities were graded as follows: 0, negative; 1, weakly positive; 2, medium-intensity positive; and 3, strongly positive. The percentage of the positively-stained cells was graded as: 0, <5%; 1, 5–25%; 2, 26–50%; 3, 51–75%; and 4, >75%. On the basis of the values that were obtained, IRS values of 0–3 were recorded as 0, 4–6 as 1+, 7–9 as 2+ and 10–12 as 3+. IRS results of 0 or 1+ were evaluated as positive and scores of 2 or 3+ were considered to be negative.

### Statistical analysis

OS was calculated as the time between the beginning of chemotherapy and mortality or the last assessment. Progression-free survival (PFS) was calculated as the time from the beginning of the treatment to progression of the disease. Disease-free survival was calculated for patients without metastases initially and was determined as the elapsed time from the first date of the gastric cancer diagnosis until the date that the disease became metastatic. For the statistical analysis, PASW Statistics for Windows Version 18 (SPSS, Inc., Chicago, IL, USA) software was used. Kaplan-Meier survival curves were drawn and a log-rank test was performed to obtain and compare the survival durations. A χ^2^ analysis was used for comparing the rates. P<0.05 was considered to indicate a statistically significant difference. If there were two comparisons with an expected value of <5, Fisher’s exact test was used.

## Results

The patients were aged between 34 and 85 years, with a median age of 58.5±15.3 years. Of the total patients, 67% (n=20) were male and 33% (n=10) were female. The median follow-up period from the occurrence of metastasis was 12.1±9.2 months (range, 2–25.3 months; 95% confidence interval, 10–17 months). The median survival duration from metastasis was 12.7±7.3 months (range, 2–25.3 months; 95% confidence interval, 9.5–16.8 months).

When the stages at the time of the application were examined, it was noted that half of the patients (n=15) were initially metastatic (16.7%; n=5) with stage IIIC diseases, 13.2% (n=4) exhibited stage IIIB disease and 6.7% (n=2) of patients were observed to have stage IIA, IIB and IIIA diseases. There were no patients with stage I disease in the present study (Table I).

### Tumor localization

The tumor was localized to the esophageal-gastric junction in 10% (n=3) of the patients, at the cardia in 13.3% (n=4), at the antrum in 23.3% (n=7), at the corpus in 23.3% (n=7) and at the corpus and antrum in 20% (n=6). However, localization was not determined in 10% (n=3).

### Pathological parameters

The parameters of the tumor pathologies, including tumor necrosis, blood vessel invasion, perineural invasion and lymphatic invasion, are presented in Table II.

### Distribution of metastases

Liver metastases were present in 36.7% (n=11) of the patients. Lung metastases were present in 13.3% (n=4). There were bone metastases present in 16.6% (n=5) and ovarian metastases in 10% (n=3) of the cases. The percentage of patients with only local recurrence was 6.6% (n=2), and brain metastases were also observed in 6.6% (n=2).

### Chemotherapy

While 13.3% of the patients (n=4) were administered only supportive care (BSC), 60% (n=18) were administered one-line, 13.3% (n=4) two-line and 13.3% (n=4) three-line chemotherapy. Frequently, docetaxel, cisplatin and fluorouracil (FU; DCF) treatment was performed in 76.7% (n=23) as the first-line chemotherapy. The other first-line treatments included epirubisin, cisplatin and FU (ECF), cisplatin and capecitabine (CX), docetaxel, cisplatin and capecitabine (DCX), docetaxel and cisplatin (DC) and tegafur uracil (UFT). While 30% (n=9) of the patients were administered second-line therapy, the treatments had been performed using FU, irınotekan and leucovorin (FOLFIRI) in 33.3% (n=3) and with capecitabine in 22.2% (n=2). Other second-line therapies were X, CF, CX, DCF and capecitabine and irinotecan (XELIRI). While the proportion of patients who were administered third-line therapy was 13.3%, these treatments had been chosen as ECF or FOLFIRI.

### EGFR status

For EGFR, the patients were grouped into those with no staining, 36.6% (n=11), those with cytoplasmic staining, 46.7% (n=14), and those with membranous staining, 16.7% (n=5). For the purposes of the analysis, the patients with cytoplasmic and membranous staining were accepted as EGFR^+^ and the patients with no staining were considered EGFR^−^. In the present study, the number of EGFR^+^ patients was recorded as 19 (63.3%).

### Survival analysis

**Figure 1:**
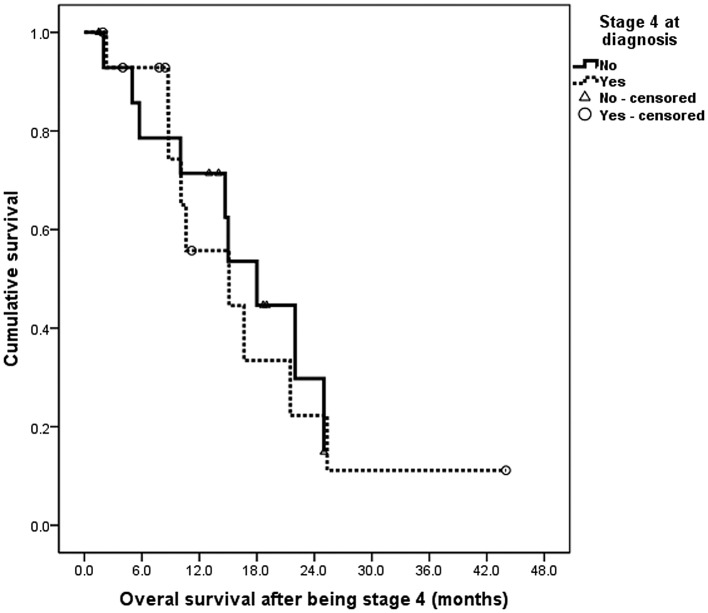
OS curves of the stage IV patients, according to the Kaplan-Meier method. Metastasis present at diagnosis vs. not present. OS, overall survival.

No differences were observed in OS between the stages of the disease. No significant differences were observed between the survival duration following recurrence in the patients with and without metastasis, between those who had undergone surgery and those who had not or between those who were administered adjuvant therapy and those who were not. The median survival time following metastasis in the patients without metastasis initially was 18 months (95% confidence interval, 12.7–23.2 months). In the patients with an initial metastatic diagnosis, the median survival time was 15.1 months (95% confidence interval, 2.8–27.4 months) and the difference between them was not statistically significant. (log-rank, P=0.841; Fig. 1).

By grouping the patients as stage IV and others, their associations were assessed using the EGFR status (Table III).

**Figure 2:**
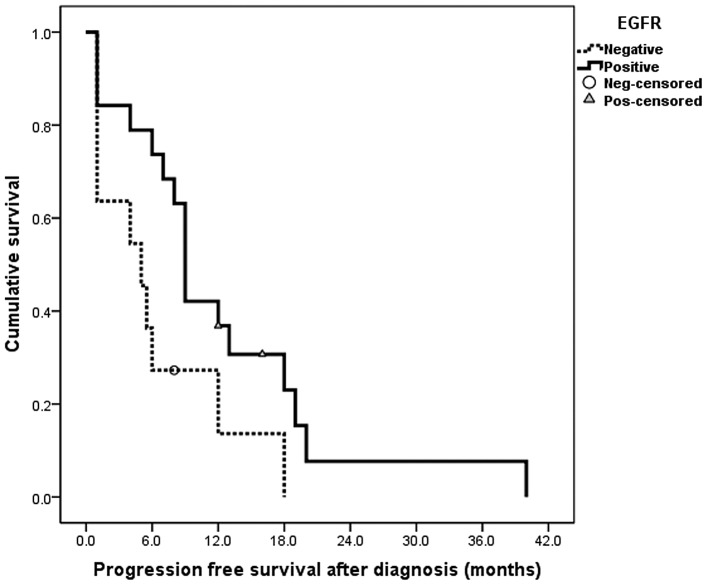
PFS curves of the EGFR^+^ and EGFR^−^ patients following diagnosis, according to the Kaplan-Meier method. PFS, progression-free survival; EGFR, epidermal growth factor receptor.

The EGFR^+^ patients tended to be diagnosed at an earlier stage than the EGFR^−^ patients. This was not statistically significant (P=0.058). Also, in the EGFR^+^ patients, a significant correlation was identified in OS (P=0.011) and PFS (P=0.039). Thus, the EGFR^+^ patients were diagnosed earlier and had an improved survival compared with those who were EGFR^−^. The median duration of PFS was five months (95% confidence interval, 0.2–9.9 months) in the EGFR^−^ patients and nine months (95% confidence interval, 7.9–10.0 months) in the EGFR^+^ patients. The difference between the two was statistically significant (log-rank, P=0.039; Fig. 2).

**Figure 3:**
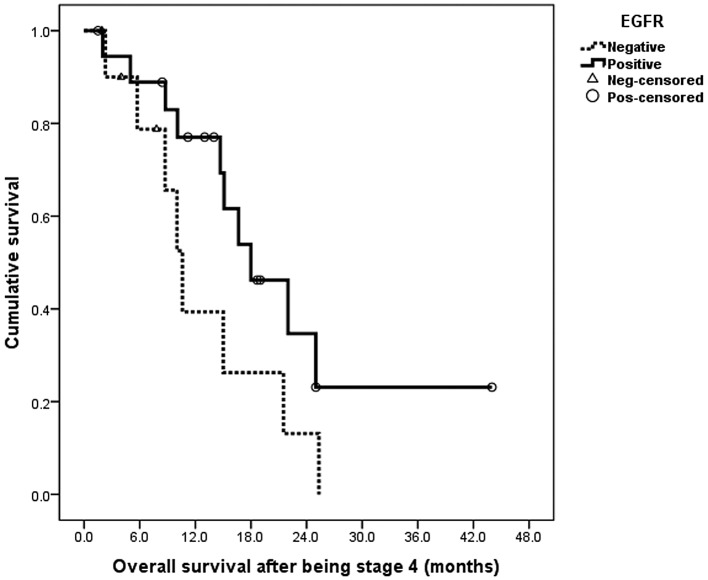
OS curves of the EGFR^+^ and EGFR^−^ stage IV patients, according to the Kaplan-Meier method. OS, overall survival; EGFR, epidermal growth factor receptor.

The median survival time following metastasis was 18 months in the EGFR^+^ patients and 10.6 months in the EGFR^−^ patients. However, this difference was not statistically significant (log rank, P=0.110; Fig. 3).

**Figure 4:**
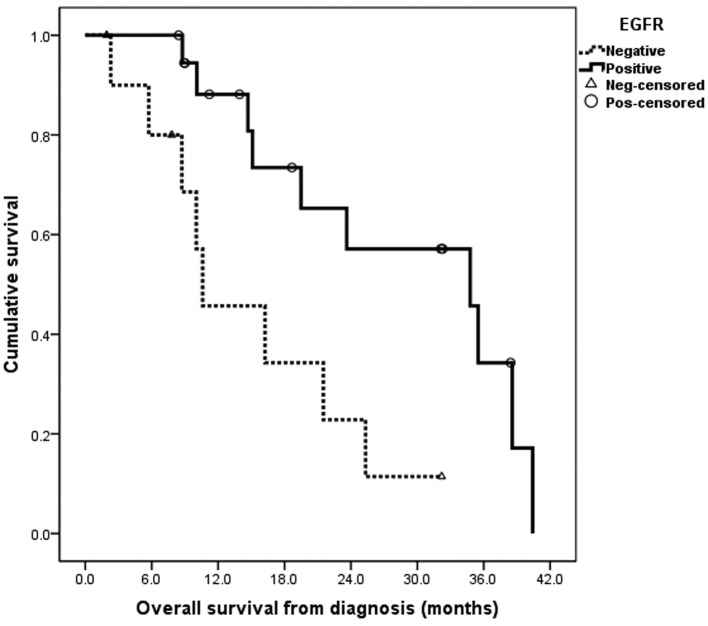
OS curves of the EGFR^+^ and EGFR^−^ patients following diagnosis, according to the Kaplan-Meier method. OS, overall survival; EGFR, epidermal growth factor receptor.

The analyses were repeated using a calculation of OS from the moment of diagnosis by including the patients that were without metastases at the time of diagnosis, regardless of the treatment of the patients. Thus, the prognosis was measured based on whether the patient was administered adjuvant treatment or not. The median survival from the time of diagnosis was 34.7 months (95% confidence interval, 19.5–49.9 months) in the EGFR^+^ patients and 10.6 months (95% confidence interval, 1.76–19.43 months) in the EGFR^−^ patients. The difference was statistically significant (P=0.01; Fig. 4).

### Chemotherapy response prediction

Generally, when the chemotherapy response (complete response, partial response and stable disease) and the EGFR status were evaluated, there was no significant correlation (Table IV).

## Discussion

In the present study, EGFR expression was determined to be 63.4%, of which 46.7% was cytoplasmic and 16.7% membranous. This rate has been reported to range from 31–74% in the literature (8,9). The main reason for this wide distribution may be the lack of a standard for the evaluation of EGFR. Although the cytoplasmic staining characteristic of EGFR has been examined in certain studies, the assessments were made according to its membranous staining status in the majority of the studies. There are fewer studies that have evaluated membranous and cytoplasmic staining together, as in the present study (9). Furthermore, technical reasons, including the differences in the kits that are used, may affect the results. The analyses were repeated according to the staining proportions in the present study, as the literature on this subject is unclear. However, the rates obtained are presented here.

In the present study, the proportion of patients with stage IV disease in EGFR negative patients were higher but statistically insignificant compared with the EGFR positive patients at the time of diagnosis (P=0.058). Also, statistically significant results were obtained for OS and PFS. The median survival times from the time of diagnosis were 34.7 months in patients with EGFR expression regardless of their stage and 10.6 months in patients without EGFR expression (P<0.01). The association between EGFR expression and prognosis in the literature is conflicting. Although it is associated with a poor prognosis in numerous studies (10–16), there are also studies that have shown EGFR expression to be associated with a good prognosis (17–19). The survival rate of 89 patients with post-operative gastric adenocarcinoma with membranous EGFR staining was shown to be worse than for others by Gamboa-Dominquez *et al* in 2004 (12).

EGFR expression using IHC and gene amplification using fluorescence *in situ* hybridization (FISH) were examined in patients with gastric cancer without distant metastases who had undergone a D2 dissection and had been administered adjuvant cisplatin and 5FU by Kim *et al* in 2009 (17). EGFR expression was observed to be positive in 80.7% of patients with IHC, but this rate was identified to be only 14% with FISH. There was no correlation between the expression of EGFR and FISH positivity. Also, in this study, as in the present study, positive correlation was not detected between the clinicopathological features of the patients, including EGFR expression and age, gender, stage or performance status. In the multivariate analysis of the present study, it was shown that low EGFR expression was an independent biomarker predictive of a shorter OS and relapse.

Furthermore, the prognosis of Chinese patients with familial gastric adenocarcinoma was investigated in a study by Ye *et al* that was published in 2011 (18). In this study, although EGFR expression in patients with gastric carcinoma was 49% (40/81), in sporadic patients it was 30.9% (25/81). EGFR rates were identified to be significantly higher in familial patients (P=0.011). Also in subgroup analyses, the five-year survival rates were lower in the familial patients with no EGFR expression (45 vs. 61%; P=0.023).

In a study by Matsubara *et al* in 2008 (15), EGFR mRNA gene expression levels in patients with advanced gastric cancer were examined. In this study, it was determined that the median time until progression for those who showed a higher level of EGFR expression was longer when evaluating patients who were administered S1 as the first-line treatment. Although the median time until progression was 2.8 months in patients who showed less EGFR expression, it was 5.3 months in patients who showed more EGFR expression. However, significant results were not detected in the patients who were administered a cisplatin-based regimen for first-line treatment. In the present study, the EGFR status was evaluated using IHC and the prognosis of the patients with positive EGFR expression was good regardless of their treatments.

Recently, the evaluation of the prognostic significance of EGFR expression in gastric cancer using membranous staining has also been investigated by Atmaca *et al*(19). EGFR positivity was not identified to be a prognostic factor in this study, which evaluated 457 patients, or in another study by Song *et al*(20).

Trastuzumab, which is a targeted therapy that is added to chemotherapy in patients with advanced gastric cancer, has been shown to be useful for treating HER2^+^ patients by the TOGA study (5). In this study, longer survival times were observed than were expected in the patient group, which was administered only chemotherapy. This may have been due to HER2 positivity. EGFR, which is a member of the same family of receptor tyrosine kinases, may similarly be a good prognostic marker in advanced gastric cancer. The prognostic and predictive role of EGFR expression remains controversial and contrasting opinions have been proposed in the literature. Differences in the studies may be due to the lack of a standard evaluation of EGFR expression in patients with gastric cancer.

Although the impact of the present study was low due to a small sample size, a positive correlation was identified between EGFR expression and survival. Determining the prognostic parameters of new treatment strategies is significant for a gastric carcinoma patient group that has a relatively low five-year survival rate. In this disease, the use of targeted molecules in the appropriate patients may increase the survival rates. In colon and lung malignancies, receptor-dependent proteins, including tyrosine kinases, and RAS status are extremely important, rather than receptor expression. For these reasons, identifying the association between the signal transduction pathways during the development, progression and metastasis of gastric cancer may lead to the development of new therapeutic strategies that may be used against these targets.

## Figures and Tables

**Table I:** Distribution of the stage status at diagnosis.

Stage	n (%)
IIA	2 (6.7)
IIB	2 (6.7)
IIIA	2 (6.7)
IIIB	4 (13.2)
IIIC	5 (16.7)
IV	15 (50.0)

**Table II:** Pathological parameters.

Parameter	Yes, n (%)	No, n (%)	Undetermined, n (%)
Tumor necrosis	6 (20.0)	6 (20.0)	18 (60.0)
Blood vessel invasion	9 (30.0)	8 (26.7)	13 (43.3)
Perineural invasion	13 (43.3)	4 (13.3)	13 (43.3)
Lymphatic invasion	13 (43.3)	3 (10.0)	14 (46.6)

**Table III:** EGFR status association with stage.

	Stage		
		
Status	Others	Stage 4	Total
EGFR^−^	3	8	11
EGFR^+^	12	7	19
Total	15	15	30

P=0.058. EGFR, epidermal growth factor receptor.

**Table IV:** Association between chemotherapy response and EGFR.

	EGFR		
		
	Negative	Positive	Total
No response	4	3	7
Response	7	16	23
Total	11	19	30

P=1.0. EGFR, epidermal growth factor receptor.
